# To what extent are adverse events found in patient records reported by patients and healthcare professionals via complaints, claims and incident reports?

**DOI:** 10.1186/1472-6963-11-49

**Published:** 2011-02-28

**Authors:** Ingrid Christiaans-Dingelhoff, Marleen Smits, Laura Zwaan, Sanne Lubberding, Gerrit van der Wal, Cordula Wagner

**Affiliations:** 1 EMGO+ Institute for Health and Care research, Department of Public and Occupational Health, VU University Medical Center, Van der Boechorststraat 7, 1081 BT Amsterdam, The Netherlands; 2 NIVEL, Netherlands Institute for Health Services Research, PO Box 1568, 3500 BN Utrecht, The Netherlands

## Abstract

**Background:**

Patient record review is believed to be the most useful method for estimating the rate of adverse events among hospitalised patients. However, the method has some practical and financial disadvantages. Some of these disadvantages might be overcome by using existing reporting systems in which patient safety issues are already reported, such as incidents reported by healthcare professionals and complaints and medico-legal claims filled by patients or their relatives. The aim of the study is to examine to what extent the hospital reporting systems cover the adverse events identified by patient record review.

**Methods:**

We conducted a retrospective study using a database from a record review study of 5375 patient records in 14 hospitals in the Netherlands. Trained nurses and physicians using a method based on the protocol of The Harvard Medical Practice Study previously reviewed the records. Four reporting systems were linked with the database of reviewed records: 1) informal and 2) formal complaints by patients/relatives, 3) medico-legal claims by patients/relatives and 4) incident reports by healthcare professionals. For each adverse event identified in patient records the equivalent was sought in these reporting systems by comparing dates and descriptions of the events. The study focussed on the number of adverse event matches, overlap of adverse events detected by different sources, preventability and severity of consequences of reported and non-reported events and sensitivity and specificity of reports.

**Results:**

In the sample of 5375 patient records, 498 adverse events were identified. Only 18 of the 498 (3.6%) adverse events identified by record review were found in one or more of the four reporting systems. There was some overlap: one adverse event had an equivalent in both a complaint and incident report and in three cases a patient/relative used two or three systems to complain about an adverse event. Healthcare professionals reported relatively more preventable adverse events than patients.

Reports are not sensitive for adverse events nor do reports have a positive predictive value.

**Conclusions:**

In order to detect the same adverse events as identified by patient record review, one cannot rely on the existing reporting systems within hospitals.

## Background

For hospital managers and healthcare providers involved in patient safety issues it is important to have access to patient safety data to facilitate decisions on interventions aimed at improving the quality and safety of hospital care. Ideally there is real-time information about patient safety, capturing incidents that reflect actual or potential risks of adverse events. An *adverse event *is commonly defined as an unintended injury that results in temporary or permanent disability, death, or prolonged hospital stay and is caused by healthcare management rather than by the patient's underlying disease process [1].

Many countries have performed retrospective patient record review studies to identify adverse events in their hospitals [2-9]. Patient record review is believed to be the most useful method for estimating the rate of adverse events among hospitalised patients [10]. However, the method has some practical disadvantages: it is time-consuming, labour intensive and expensive [11]. Moreover, retrospective record review does not provide real-time information. It is often not possible to gain additional information about the events from the people involved.

Hospitals would benefit from reporting sources, which can provide information on patient safety periodically and on demand. The value of incident reporting by healthcare professionals has been widely recognised. Moreover, it is increasingly believed that patients (or their relatives, hereafter together named "patients") can play an important role in signalling safety issues. In the report of the UK Department of Health "An organisation with a memory" [12], the importance of both sources is stressed.

So far there is little knowledge about the comprehensiveness of incident reporting systems in hospitals. A general weakness that is often mentioned in literature about incident reporting by healthcare providers is that incidents are considerably under-reported [13,14]. A few studies compared results from record review with either incident reports or patient complaints in small scale study designs [13,15,16]. They found little overlap in events detected by the methods they compared and concluded that incident reporting or patient complaints alone do not provide an adequate assessment of adverse events. As far as we know, no earlier studies have reported on the completeness of both incident reporting by healthcare professionals and reporting by patients (complaints as well as claims) in regard of detecting adverse events.

The aim of our study is to get insight into the extent in which four hospital reporting systems of incidents reported by healthcare professionals and complaints and claims reported by patients cover adverse events that were identified by patient record review. We want to know if the diverse reporting systems, which are already implemented in Dutch hospitals, are individually or cumulatively useful as a method for identifying adverse events (see Box 1 for some background information about incident reporting by healthcare professionals and the procedures for complaints/claims by patients in the Netherlands).

Moreover, we are interested whether adverse events with a higher preventability and severity of consequences have a larger likelihood of being reported. It is our assumption that highly preventable adverse events might be reported relatively often, because the persons involved feel the hospital can learn from these events to prevent them in the future. Severe adverse events might have an increased chance of being reported, because of their visibility and impact on the patient. When highly preventable and severe adverse events are regularly reported in one or more reporting systems, the issue of under-reporting is less worrying than is stated in literature.

Our study has four specific research questions: 1) How many adverse events established by patient record review are also identified in one or more reporting systems of incident reports, complaints and claims? 2) What is the amount of overlap in the detection of adverse events between the reporting systems? 3) Is the degree of preventability and severity of adverse events related to the likelihood they are reported? 4) What is the sensitivity and specificity of reports for adverse events?

### Incident reporting, complaints and claims in Dutch hospitals

In the Netherlands, healthcare professionals have been increasingly stimulated to report incidents or near misses within their own hospital. For the healthcare professional in 2004 reporting incidents (especially adverse events and calamities) was not mandatory by law but a consequence of healthcare quality and patients' rights legislation. It often was mandatory by employment contract. The incident reporting system is by definition meant for all incidents, including adverse events. An incident can be defined as "Any deviation from usual medical care that causes an injury to the patient or poses a risk of harm. Includes errors, preventable adverse events, and hazards." A near miss is defined as "Serious error or mishap that has the potential to cause an adverse event but fails to do so because of chance or because it is intercepted." [17]. This means that an adverse event is always an incident but an incident does not has to be an adverse event. After an incident/near miss has happened, it can be reported by filling out an electronic or paper-based report form containing, among other things, a description of the event, the time and place of occurrence and the people involved. The incident reporting committee will register and analyse the incidents.

Patients are encouraged to report their complaints without further definition.

In the Netherlands patients have various options to file a complaint or claim in the Netherlands when they are not satisfied with the care or cure they received. The choice of the path depends largely on the intention of the patient making the complaint or claim.

There are different reasons for patients to complaint, for example, patients want to have an explanation or an apology or initiate an investigation on the legitimacy of certain acts committed. Reasons to file a claim are for example to get financial compensation or to prevent recurrence of the incident to restore their sense of justice [18]. Patients can submit their grievances to the complaints officer or to the more formal complaint committee within the hospital. The purpose of an informal complaint is mediation or expressing a concern about the quality of care, whereas a formal complaint is made to instigate an investigation followed by a formal judgement about the legitimacy of the complaint (not juridical binding). A legal option outside the hospital is to submit a formal appeal to the medical board to obtain a verdict or when a financial compensation is wanted, to file a claim to the hospital board. The Netherlands does not have a no-fault system. Malpractice claims are judged by the insurance company of the hospital. If patients do not agree with the judgement of the insurance company on the liability or the financial compensation, patients can approach a civil court. It is possible for patients to use more than one path simultaneously or consecutively. Reporting systems are not set up with the intention to report adverse events in specific. Patient reports often concern the quality of care, whereas healthcare professionals frequently report about deviations from procedures.

## Methods

### Study design and setting

A retrospective study was performed using a database of reviewed patient records collected during a retrospective patient record review study examining the incidence of adverse events [1,9]. This record review study was conducted in 21 hospitals, involving 20% of the acute care hospitals in the Netherlands. All 21 hospitals were invited to participate in our supplementary study on the completeness of incident reporting systems. Fourteen hospitals were willing to participate: two university hospitals, four tertiary teaching hospitals and eight general hospitals. Hospital sizes ranged from 201 to 985 beds. Seven hospitals declined because of practical reasons, such as simultaneous participation in other projects, time constraints, elimination of the reporting system after two years or having an anonymous reporting system (no patient information registered).

Four reporting systems per hospital were linked with the adverse events database in these fourteen hospitals: 1) the reporting system of the complaint officer: for informal complaints by patients; 2) the reporting system of the complaint committee: for formal complaints by patients; 3) the reporting system of the liability officer: for medico-legal claims by patients and 4) the reporting system of incident reports: for incident reports by healthcare professionals. For each adverse event identified in the patient records, the equivalent was sought in the four reporting systems of the same hospitals by comparing the date and descriptions of the events.

This study is a continuation of the Dutch patient record review study [1,9], for which ethical approval was granted by the VU University Medical Center in Amsterdam. The participating hospitals formally consented to take part in this study.

### Patient record review

In each hospital a stratified random sample was selected of 200 admissions of patients discharged from the hospital in 2004 (> 24 hours stay) and 200 (or less if the total of patients who died in 2004 was lower) admissions of patients deceased in the hospital in the year 2004, excluding admissions of psychiatry, obstetrics and children less than one year old.

Between August 2005 and October 2006, 55 trained physicians reviewed the medical, nursing and, if available, the outpatient record of all sampled admissions that contained triggers for adverse events, for example an unplanned readmission, unplanned return to the operating room or unexpected death. The presence of one or more of the 18 predefined triggers was judged in advance by trained nurses. For each patient record two physician reviewers determined independently the presence of one or more, consequences, and degree of preventability of the adverse events, based on a standardised procedure and review form. A *preventable *adverse event was defined as an adverse event resulting from an error in management due to failure to follow accepted practice at an individual or system level. Accepted practice was taken to be 'the current level of expected performance for the average practitioner or system that manages the condition in question'. The degree of preventability was measured on a six-point scale from "(Virtually) no evidence of preventability" to "(Virtually) certain evidence of preventability".

The degree of severity of the consequences was rated on a seven-point scale from "No physical impairment or disability" to "Death". The methods of determining adverse events were based on the well-known protocol of The Harvard Medical Practice Study and have been described in detail elsewhere [1].

### Linking record reviews with reporting systems (finding patient identity matches)

Between March 2007 and March 2008 fourteen hospitals provided datasets from each of the four reporting systems, containing identification characteristics of all patients involved in an incident report, complaint or claim in the year 2004: patient registration number, date of birth and sex and hospital. Reports without sufficient patient identifiers or reports of events which occurred in 2003 but were reported in 2004 were non-eligible for matching. Because of the confidentiality of the information, the researchers had no access to the reporting systems in the hospitals. Sometimes exceptions were made when the incident reports, complaints or claims were not digitally registered. In these cases, the researchers got permission to access the reports to get the information required. A confidentiality agreement was signed by the researchers to maintain secrecy of the information. Data from the reporting systems were recorded anonymously and all information was kept confidential.

**Figure 1:**
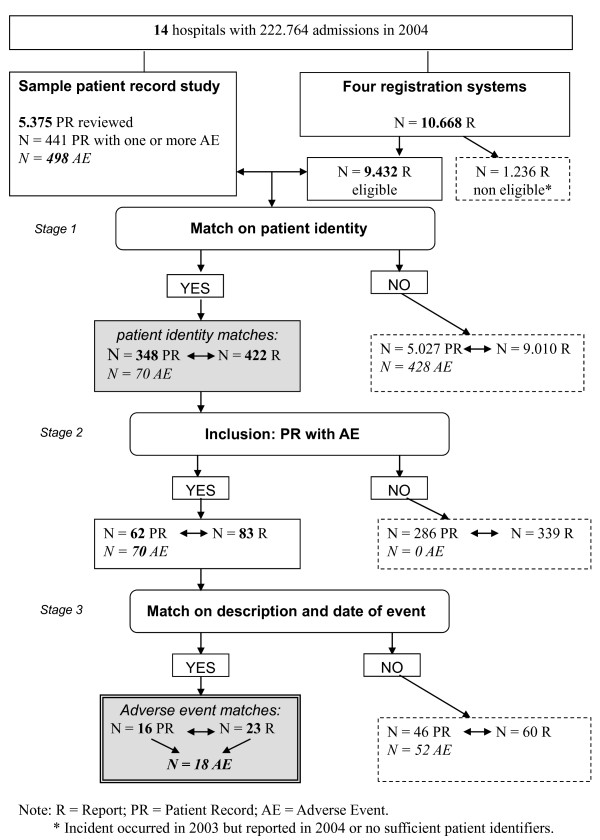
**Flow chart of study procedure**.

The patient identification data from the four sources were then linked with the database of the sample from the record review. A case matched when a patient involved in the complaint, claim or incident report matched on identity with a patient from the record review sample. These matches were classified as *patient identity matches *(see Figure 1).

### Comparison of narratives (finding adverse event matches)

It is possible that a complaint, claim or incident report - hereafter together named "reports"- concerned another issue than the adverse event identified by record review. For example, relatives of a patient complaining about how the doctors communicate with the patient and not about the adverse event that involved a wrong dose of medication. To be able to examine whether the identity matches related to the same issue, we obtained more information about these events, including the original description of the event, the date of occurrence and consequences for the patient, by means of a questionnaire.

Furthermore we obtained information about the characteristics of the reporter (patient or healthcare professional). The questionnaires were sent to the officer of each reporting system in the hospitals. A researcher (ICD) compared the dates and descriptions of the adverse events from the record reviews with the dates and descriptions/narratives of the reports. The descriptions of the events did not need to correspond perfectly. The physicians who reviewed the patient records made a medical description of the adverse event, while patients generally do not use professional language when describing their dissatisfaction. Moreover, an adverse event can evolve from a chain of events in time, while a report often describes just a part of the situation. Allowing for the above mentioned differences, cases were classified as a match if it was plausible that: 1) the descriptions were about the same event or 2) the description in the report was about an event that was related to the adverse event (for example, the adverse event was a consequence of the reported event). The comparisons were discussed with other researchers (LZ, CW). In doubt and for the final verification a physician was consulted (MD).

Matched cases in this final stage-meaning that the topic of the report was related to an adverse event in the record review study-were classified as *adverse event matches *(see Figure 1).

### Statistical analysis

Usefulness of the reporting systems for detection of adverse events was determined by the number of adverse event matches (absolute number and percentage of all possible matches). The extent of overlap between reporting systems was determined by counting the number of adverse events that were detected in more than one reporting source. The characteristics of the reporters of each report were described in percentages of the following categories: professionals (physician, resident/physician, nurse and student nurse) and patients (patient, child, spouse, child & spouse and legal adviser).

To test whether the degree of preventability and severity of the adverse events influence the likelihood they are reported, we performed t-tests. Results were considered statistically significant at p < 0.05. For the non-significant differences we calculated the power. Data were analysed using SPSS 15.0.

Each patient was identified as being positive or negative for an adverse event for one or more reports that matched an adverse event in the patient record review. Sensitivity and specificity interpret the reports results retrospectively whereas positive predictive values and negative predictive values establish the predictive properties of the reports in the future.

## Results

In the record review study in 14 hospitals, a total of 5.375 patient records were reviewed. The reviewers identified 498 adverse events. In a few medical cases more than one adverse event was identified. A flow chart of the study process is presented in Figure 1.

The fourteen hospitals received in total 10.668 reports in four reporting systems in 2004. Of these reports 1.236 reports were not eligible, because they were anonymous, not related to a specific patient or the report concerned an event that occurred in 2003. For our study 9.432 reports were eligible: 5.592 incident reports, 3.384 informal complaints, 186 formal complaints and 270 medico-legal claims.

Since 498 adverse events were identified in the patient record study, patients and healthcare professionals in theory could have reported 498 adverse events as a complaint, claim or incident report in one or more reporting systems. After matching on patient identifiers in stage 1, an overlap was found between 422 (of 9.432) reports of patients and healthcare professionals and 348 (of 5375) patient records (patient identity matches).

Healthcare professionals reported 353 incidents and patients submitted 61 informal complaints, 5 formal complaints and 3 medico-legal claims. Sometimes a patient was identified in more than one reporting source.

Due to confidentiality agreements we could not disclose the patient records in which the adverse events were detected. Therefore we send the officers of the reporting committees a questionnaire for each report that matched on patient identifiers with a patient record for more information about these reports (n = 422 questionnaires). The response rate was 100%.

In stage 2 we excluded the patient records in which no adverse events were detected and the reports that matched on identity with these patient records.

**Figure 2:**
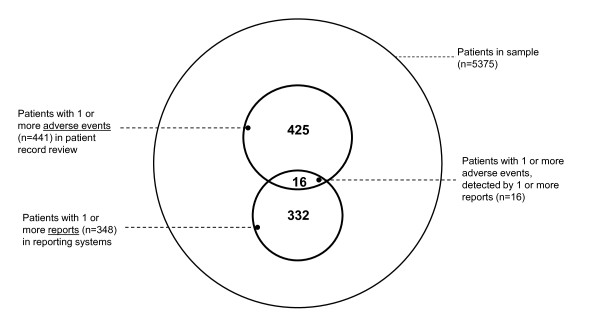
**Degree of overlap in patients with AEs between patient record review and reporting systems (n = 5375)**.

The patient identity matches now involved 62 patient records with 70 adverse events in the patient record review and 83 reports in the reporting systems. Comparison of the content of the 83 reports with the 70 adverse events in stage 3 showed that 18 adverse events of the patient record review study were reported in one or more reporting systems (adverse event matches); that is 3.6% (18/498) of all possible adverse event matches. (The result of the matching on adverse event identity is presented in Figure 2.)

**Figure 3:**
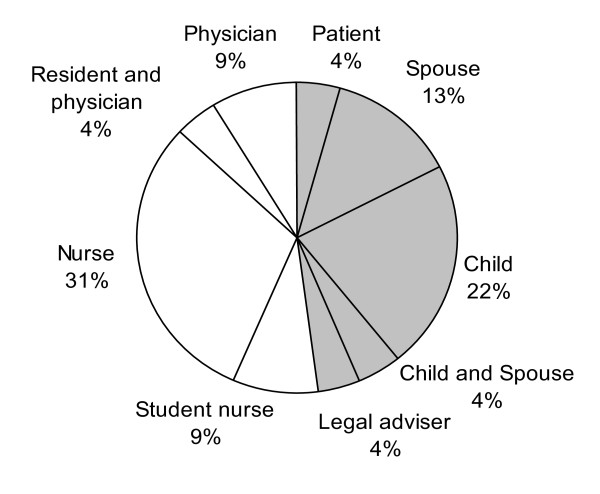
**Characteristics of reporters (n = 23 reporters/reports)**. Characteristics of reporters of each report that matched with a patient record review on patient identifier and AEs.

Healthcare professionals reported 10 adverse events in 12 reports (2.0% of all possible adverse event matches). Patients reported 14 adverse events in 11 reports (2.8% of all possible adverse event matches). The characteristics of the reporters show that (student) nurses made most of the adverse event reports (40% of all reports) (Figure 3). On behalf of the patient, in the majority of complaints the patients' children were involved (26% of all reports).

**Figure 4:**
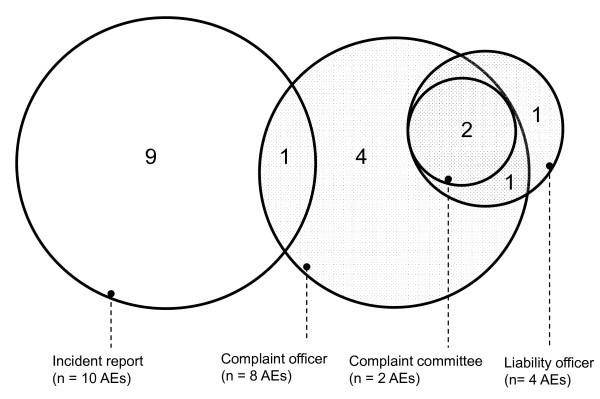
**Degree of overlap of AEs between registration sources (n = 18 AEs)**.

There was only a small overlap in reporting systems (Figure 4). One adverse event was identified in both a patient complaint and an incident report. In another case a patient used two sources to complain and submit a claim (complaint officer and liability officer) and in two cases three sources were used (complaint officer, complaint committee and liability officer).

The mean preventability of adverse events that were reported was 3.61 (n = 18; SD = 1.72) compared to 3.01 (n = 480; SD = 1.72) of those that were not reported in any of the reporting systems (no significant difference, power = 30.6%). The mean preventability of reported adverse events was higher for events reported by healthcare professionals (mean = 4.60; n = 10; SD = 1.07) than events reported by patients (mean = 2.38; n = 9; SD = 1.60), (p < 0.05).

The mean severity of adverse events that were reported was 5.33 (n = 18; SD = 2.24) compared to 4.49 (n = 480; SD = 2.45) of those that were not reported (no significant difference, power = 34.4%). The mean severity of reported adverse events was higher for events reported by patients (mean = 6.13; n = 9; SD = 1.64) than for events reported by healthcare professionals (mean = 4.70; n = 10; SD = 2.54), although not statistically significant (power = 31.3%).

The sensitivity and positive predictive value of the reports for adverse events was 3.6% and 4.6%. The specificity was 93.3% and the negative predictive value was 91.5%

## Discussion

### Main findings and interpretation

Among the 498 adverse events identified by patient record review only 18 adverse events (3.6%) were identified by record review, meaning that the majority of 480 adverse events was not registered in one of the four registration systems.

Most of the adverse events (n = 17) were detected in either a professional's report (n = 9) or a patient's report (n = 8); only one adverse event was detected in both systems. Patient reports and healthcare professional reports contributed almost equally to the detection of adverse events. Since healthcare professionals overall made over five times more reports than patients did, we can conclude that there is a higher chance to find an adverse event in reporting systems of patient reports than in incident reporting systems for healthcare professionals. In incident reporting systems for professionals, most of the reports are made by nurses and, therefore, probably mainly concern nursing care. Adverse events, in contrast, often concern medical care provided by resident physicians and medical consultants. The fact that these professionals are the ones making few reports in reporting systems could explain the relatively low number of adverse event matches in incident reports. Patients, on the other hand, report about the whole sequence of care. Generally, both healthcare professionals and patients report about a broader group of incidents than mere adverse events. Often, their reports concern quality issues without patient harm.

Statistical calculations showed that the degree of preventability and severity of the adverse events does not influence the likelihood they are reported. The results showed no statistically significant differences. However the non-significance might be the result of the low power.

Patients and healthcare professionals reported relatively more preventable and severe adverse events compared to less preventable and severe adverse events. The degree of preventability of adverse events reported by healthcare professionals was relatively higher than of adverse events reported by patients. We found that healthcare professionals reported more preventable adverse events and patients more severe adverse events. The learning potential of the adverse event or its visible impact on the patient might contribute to reporting behaviour. But, unfortunately, not all highly preventable and severe adverse events are registered.

Because of the low number of adverse events detected in the reporting systems, under-reporting of especially preventable and severe adverse events remains to be a problem.

Patients made only a few medico legal claims. In the Netherlands, there is not a real claim culture. In addition, patients can only file a claim when they are informed about the possibility to financially redress the harm that is suffered.

On the whole, the results show that in order to detect the same adverse events identified by record review, one cannot rely on the existing reporting systems within hospitals at present.

Why are so few adverse events reported by healthcare providers or patients? Healthcare professionals might be embarrassed or afraid of condemnation by their colleagues or the hospital management after reporting adverse events they were involved in, especially those with severe consequences. Furthermore, there are other barriers mentioned in literature that influence event reporting, including: fear of disciplinary action or potential litigation, time pressure, no feedback, the perception that reporting is unnecessary, unclear reporting procedures and a lack of clear definitions as to what constitutes a reportable event [19-25]. Moreover, resident physicians and medical consults do not generally perceive (surgical) complications to be "reportable incidents". They address complications, among other incidents, in Mortality and Morbidity meetings (M&M) [26]. There are also a number of possible reasons why patients do not report adverse events [19]. Patients may not be aware they have sustained harm from medical care, while it is not easy to disentangle medical injury from the development of the underlying illness. Moreover, patients can be unaware of the possibilities of making a complaint or claim. Or they can be unwilling to do so, because they do not want to commit the time and energy needed to take action, they are concerned the report will bring tension into the relationship with their doctor or they do not feel the need to complain, because their doctor clearly explained the event to their satisfaction (disclosure). Moreover patients might not feel the need to file a claim because in the Netherlands the healthcare and social security systems are well developed and patients do not feel the need for financial compensation.

Finally, patients with grievances or concerns may choose to speak directly to their healthcare provider. Other patients that have experienced an adverse event can choose to step outside the hospital and submit an appeal to e.g. the medical disciplinary board or civil court.

The sensitivity and positive predictive value is low (3.6% and 4.6%), meaning that reports of patients and healthcare professionals are not useful as a predictive method to detect the same adverse events as a patient record study. Although specificity and negative predictive value seem high (93.3% and 91.5%), one has to bear in mind that an absence of a report does not imply that no adverse event occurred. We did not researched the types and consequences of the reports, therefore it might be possible that reports that did not match still concerns adverse events.

Although incidents, complaints and claims do not detect the same (number of) adverse events as the patient record review, reports can still be useful in identifying issues concerning patient safety. In the sample 441 patients were involved in one or more adverse events and 348 patients were involved in one or more report. With an overlap of 16 patients 332 patients were still involved in situations that healthcare professionals or patients found important enough to report.

### Comparison with previous research

Different from our study design, other studies compared adverse events identified with record review with either incident reporting by healthcare professionals or patient complaints [13,15,16]. Olsen et al. compared local real-time record review with incident reporting and pharmacist surveillance [15]. As in our study, the authors found little overlap in events detected by the different methods. In a study by Sari et al., data of record review were compared with data submitted to a routine incident reporting system of the same patients [13]. They found that the reporting system missed most patient safety incidents that were identified by record review and detected only 5% of those incidents that resulted in patient harm (these incidents probably included adverse events). In our study, 2% of the adverse events that were identified by record review were reported by healthcare professionals. The authors of both studies concluded that incident reporting alone does not provide an adequate assessment of adverse events and recommend hospital staff and researchers to use more than one method at the same time [13,15].

Bismark et al. compared results from a record review study with patient complaints: 0.4% of the adverse events resulted in complaints [16]. In our study, 1.8% of the adverse events were detected in complaints and claims. Bismark found that the odds of a complaint were higher for adverse events with higher preventability and severity [16]. Because of the low numbers we could not compare the results.

### Strengths and limitations

No earlier study has compared record review with both registration systems of reports by healthcare professionals and patients (complaints as well as medico-legal claims). We studied a large number of adverse events and included multiple hospitals in our study design, increasing the likelihood that our results can be generalised to other hospitals.

Our study has, however, several limitations. Firstly, complaints and claims relating to episodes of care in 2004 may have been submitted later, outside our study period (January-December 2004), especially those that are submitted to the Complaint Committee and Liability Officer. Moreover, in some hospitals resident physicians and medical consultants also make reports of adverse outcomes of medical care during Morbidity and Mortality meetings (M&M). It was not possible to include this as a source in our study, because in some hospitals M&M reports are written down on notepads and are not formally registered. In other hospitals, there was only an M&M registration for surgery or not an M&M registration at all. Finally, patient record review has recognized limitations regarding the estimation of adverse event rates [27,28]. Thomas et al. stated that estimates of adverse events rates from patient record review are highly sensitive to the degree of consensus and confidence among reviewers [28]. Therefore it is possible that reports contained adverse events that were not identified in the record review study. Because of these limitations, the number of adverse events that can be detected by patients and healthcare professionals might be underestimated.

## Conclusions

An examination of reports from healthcare professionals and patients in reporting systems is not sufficient to detect a substantial number of adverse events and thus cannot replace record review. Adverse events are seriously under-reported. Barriers of reporting should be reduced.

The results show that there is little overlap in adverse events covered by healthcare professionals' and patients' reports: both groups reported different adverse events. There is underreporting of adverse events by both groups, but using either reports of professionals or patients would have yielded even fewer adverse event matches.

Considering the large numbers of patients' and healthcare professionals' reports that were not related to an adverse event, incident reporting systems, complaints and claims could, however, carry a vast amount of valuable information on the quality of care that can be used for the improvement of hospital healthcare. In future research, it seems worthwhile to study what information these sources can offer regarding the quality and safety of hospital care.

## Competing interests

The authors declare that they have no competing interests.

## Authors' contributions

ICD contributed to the design of the study, coordinated the data collection, analysed and interpreted the data and drafted the manuscript. MS wrote the manuscript and contributed to the analysis and interpretation of the data. LZ contributed to the data analysis and critically read the manuscript. SL contributed to the data collection and critically read the manuscript. GvdW and CW contributed to the design of the study and interpretation of the data, and revised the manuscript critically for important intellectual content. All authors gave their approval of the final version of the manuscript.

## Pre-publication history

The pre-publication history for this paper can be accessed here:

http://www.biomedcentral.com/1472-6963/11/49/prepub
